# The provision of healthcare services to older LGBT adults in the Nordic countries: a scoping review

**DOI:** 10.1080/02813432.2023.2242713

**Published:** 2023-08-21

**Authors:** Monika Dybdahl Jakobsen, Janne Bromseth, Anna Siverskog, Martin Sollund Krane

**Affiliations:** a Centre for Care Research North, UiT The Arctic University of Norway, Tromsø, Norway; b Eastern Norway Research Institute, Hamar, Norway; c Department of Culture and Education, Södertörn University, Huddinge, Sweden

**Keywords:** Healthcare services, older LGBT adults, the Nordic countries, scoping review, education of healthcare professionals

## Abstract

**Objectives:**

Our objectives were to examine what is known about the provision of healthcare services to older LGBT adults in the Nordic countries, identify knowledge gaps, map implications of this research for the education of healthcare professionals and delivery of healthcare, and identify key future research priorities to advance policy and practice for older LGBT adults in this region.

**Design:**

We conducted searches in nine databases. Peer-reviewed articles and PhD theses published in and after 2002 written in English, Norwegian, Swedish or Danish languages were included. 41 studies met our inclusion criteria. However, only eight of these studies focused specifically on older LGBT adults. Therefore, to answer all research questions, five book chapters about older groups were also included.

**Results:**

There were few studies from countries other than Sweden and few quantitative studies. Bisexual people represented a neglected group in research. The studies included showed that healthcare personnel lack knowledge on LGBT issues, particularly about older LGBT adults and non-binary gender identification. Older LGBT adults frequently reported being met with cis- and heteronormative expectations in healthcare encounters. For transgender people, access to medical treatment has been managed by gatekeepers influenced by a binary understanding of gender.

**Conclusions:**

Relevant measures to enhance practices are increased attention on LGBT issues in education; training of healthcare professionals; measures at the institutional level; and ensuring that transgender people identifying as non-binary receive the same quality of care as individuals identifying in a binary way.

## Abstract (plain language summary)

To advance healthcare practices for older LGBT adults in Nordic countries, it is vital to review the available knowledge.

There are few studies about *older* LGBT adults and few studies from Nordic countries besides Sweden.

Studies indicate that healthcare personnel lack knowledge on LGBT issues and that older LGBT adults are met with cis- and heteronormative expectations.

Studies suggest increased attention to LGBT issues in education, training of healthcare personnel, institutional level measures, and that transgender people who identify as non-binary receive the same quality of care as others.

## Introduction

Review articles suggest that healthcare services lack inclusiveness towards older^1^ [1] LGBT^2^ adults [2–5]. According to a review of studies in the USA, older LGBT adults experience that providers of healthcare services lack sufficient knowledge about LGBT health issues [2]. Three review articles, one which included studies from the UK [4] and two which mainly included studies from the USA and Canada [3,5], all report that older LGBT adults experience heteronormative assumptions when contacting healthcare services. Furthermore, the mentioned review articles find that older LGBT adults experience and/or fear discrimination when accessing healthcare services and conclude that heteronormativity, negative attitudes, and expected or experienced discrimination have negative consequences for LGBT users [2–5]. Some older LGBT adults have felt forced ‘back into the closet’, while others abstain from, delay, avoid or discontinue the use of healthcare services [2–5]. Such avoidance of services use may have negative health impacts.

During the last six-seven years, there has been a considerable increase in published studies concerning healthcare services to older LGBT adults, and these studies also include literature reviews. However, no scoping reviews or systematic reviews concerning healthcare services to LGBT people in the *Nordic countries* have been published. The organization of healthcare services is quite similar within the Nordic countries, and the ‘Nordic model’, built on egalitarian ideas and characterized by taxation-based universal access, differs from most other countries. In addition, the LGBT rights and level of acceptance of LGBT people rank relatively high in the Nordic region when compared to other countries, although very different attitudes towards LGBT people coexist within these countries [6,7]. Consequently, the transferability of knowledge on this topic from other parts of the world to a Nordic context is less than certain. Therefore, we find it important to assess the literature concerning the provision of services to older LGBT adults in the Nordic countries.

In this study we aimed to (a) examine what is known about the provision of healthcare services to older LGBT adults in the Nordic countries, (b) to identify knowledge gaps in the evidence, (c) to map the implications of this research for education and for delivery of services to older LGBT adults, and (d) to identify key future research priorities to advance policy and practice for older LGBT adults. The overall aim of mapping this knowledge is to contribute to the enhancement of healthcare policies and practices for older LGBT adults in the Nordic countries.

## Methods

We used a scoping review approach to assess the research literature in this field, since scoping studies are recommended in fields with emerging evidence and when the aims of the review are mapping knowledge and identifying knowledge gaps [8,9]. The scoping review was carried out in accordance with the stages outlined in the Arksey and O‘Malley framework [10] and Levac’s elaboration of this framework [9]. Consequently, we first identified research questions, and then relevant studies through systematic searches, before selecting studies (based on inclusion criteria), charting the data, and finally collating, summarizing, and reporting the results. Before beginning the study, to predefine the aims and methods of our scoping review, and to allow for transparency of the process, we developed a protocol, which was published in the Open Science Framework [11]. The present review was reported in accordance with the Preferred Reporting Items for Systematic Reviews Extension for Scoping Reviews (PRISMA-ScR) (Appendix 1) [12].

### Identifying the relevant studies

Our search strategy aimed to find studies published in peer-reviewed journals and approved PhD theses. An initial limited search of Web of Science, PUBMED, and Swemed was undertaken to identify relevant articles and search terms. We then carried out a search which included the following databases: Svemed, Sociological Abstracts, Nora Open, MEDLINE, Web of Science, Cinahl, Diva-portalen, Proquest Dissertation and Google Scholar^3^. Our search strategy, including all identified keywords and index terms, was adapted for each included database in an iterative process. The reference lists of all included sources were screened for additional studies.

Only studies published in English, Norwegian, Swedish and Danish were included, due to the authors’ lack of language skills in Finnish and Icelandic. Studies published in and after 2002 were included. This was because we considered that attitudes to LGBT people have changed in the Nordic countries recent decades [7]. These changing attitudes are reflected in legislation where gender-neutral marriage as well as anti-discrimination laws, have been implemented in the Nordic countries in the last two decades.

### Study selection

**Figure 1.**
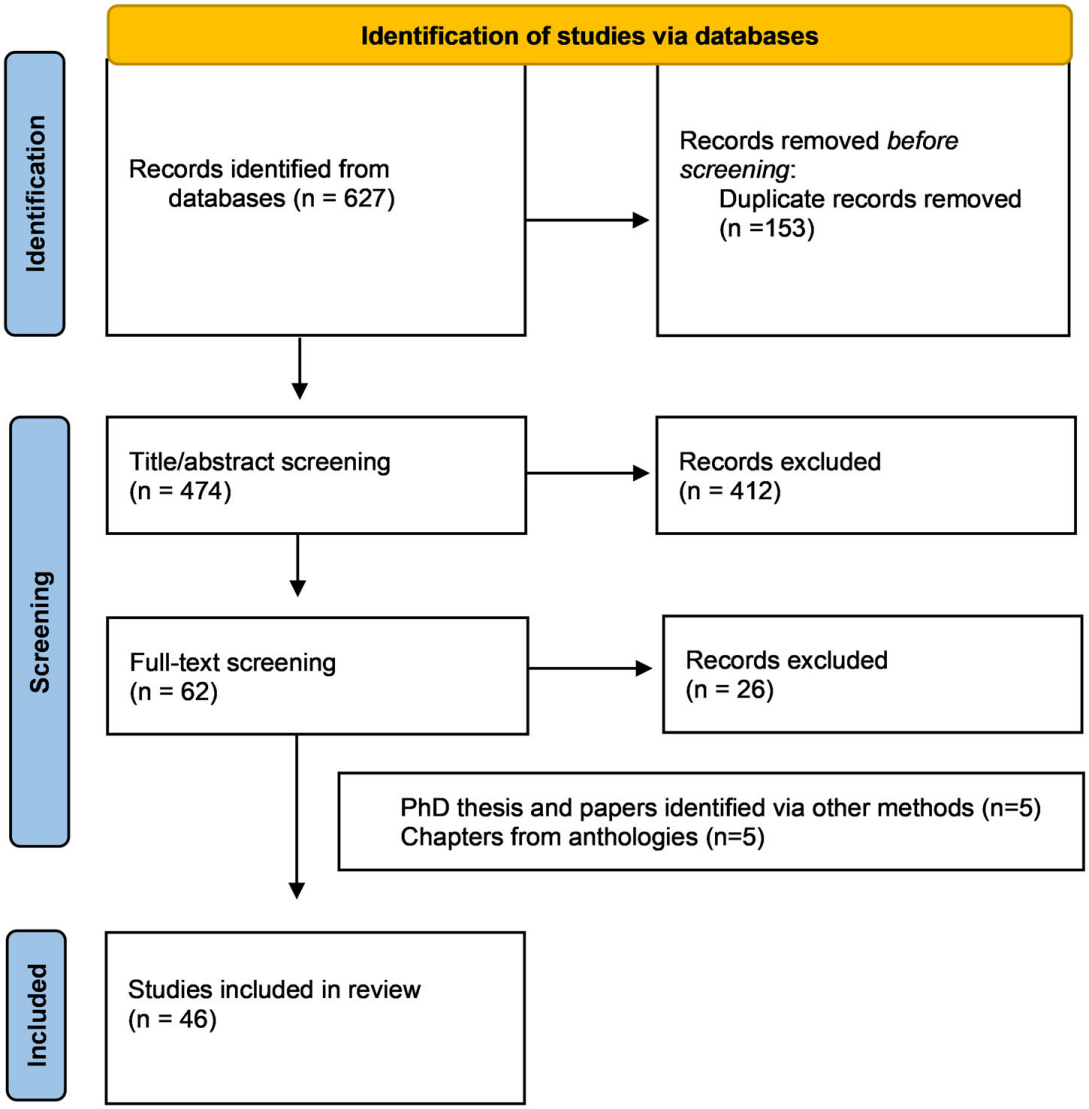
Flow diagram of the study selection process and outcomes.

The search identified 627 results (Figure 1). All identified references were collated and uploaded into EndNote 9.33 (Clarivate Analytics, USA), where duplicates were removed. All results were transferred to the RAYYAN system (*Rayan Systems Inc., USA*), where MDJ and MSK independently screened the titles and abstracts (*N* = 474) against the inclusion criteria for the review (Table 1). Then the full text of selected citations (*N* = 62) was assessed in detail against the inclusion criteria by MDJ and MSK independently. Disagreements between the two reviewers were resolved through discussion. 36 texts were identified through database searches. In addition, one paper was found through reading a PhD thesis identified through the search, and one PhD thesis and three papers were included as a result of the authors knowledge of the field.

**Table 1.** Initial inclusion and exclusion criteria.

	Inclusion criteria	Exclusion criteria
Publication type	Scientific papers published in peer-reviewed journals and approved PhD theses in English, Norwegian, Swedish or Danish. Empirical and non-empirical.	Chronicles, comments, leaders and books. Publications in Finnish and Icelandic.
Participants	Studies including at least one person living in the Nordic countries, aged 60 or older who self-identified as LGBT, people with whom they had a significant relationship, healthcare service providers and students in the Nordic countries.	
Concept	Healthcare services to LGBT people; LGBT peoples’ experiences with healthcare services; LGBT peoples’ strategies used in contact with healthcare services; healthcare professionals’ and students’ competence, attitudes and training needs concerning LGBT adults; how LGBT issues are addressed in the education of healthcare personnel.	Studies concerning LGBT health; health risk; medical topics; screening participation; effect of interventions to prevent sexually transmitted infections; HIV testing; maternity care; gender reassignment surgery techniques; prevalence and incidence studies.

Only eight of the 41 (so far) included texts focused exclusively on *older* age groups, and few of the other texts explicitly mentioned older age groups. Seven of the texts focusing on older adults were Swedish, and one Norwegian. The latter, however, only briefly mentioned healthcare services. Therefore, to include more texts concerning healthcare services to older LGBT adults from countries other than Sweden, be able to better answer the research questions, and be able to cover topics that had not been mentioned in the texts included up to that point, we added five book chapters from three anthologies, all which explicitly focused on older LGBT adults and healthcare services. These book chapters had all passed through a review process similar to peer-reviewing before publishing. Of these chapters, one was Norwegian, one was Finnish, and the remaining three were Swedish.

### Charting the data

Data from texts included in the scoping review was extracted using a data extraction tool developed by MDJ and MSK (Appendix 2). They independently charted the data from six studies to determine whether their approach to data extraction was consistent and the data-charting form was consistent with the research questions, before using the extraction tool on all included studies. The data extracted included specific details about the author(s), year of publication, country of origin, methods, respondents/data, and findings related to what is known about the provision of healthcare services to older LGBT adults and implications for education and delivery of healthcare services to older LGBT adults.

### Collating, summarizing, and reporting the results

After all texts were charted, MDJ and MSK independently read through all data extraction tools and noted central results relevant to the research questions, before both authors’ results were summarized on large sheets. Thematic categories found during this process were *lack of knowledge; attitudes; education; cis- and heteronormativity; structural factors; social network; partners; sexual practice; and gender-affirming medical treatment*. The results were then reported in a paper draft. During this writing process, parts of the original texts were reread. Subsequently, JB and AS read the draft, paying particular attention to whether important topics were left out or were inaccurately presented.

## Results

### Characteristics of included studies

As previously mentioned, 41 texts (36 articles and five PhD theses) met our original inclusion criteria, and five book chapters from three anthologies were also included. Thus, a total of 46 texts were included in the review. Four of the five PhD theses were based on papers that also were included in our search. Analysis showed that a clear majority of the studies (80%; 37 texts) were from Sweden. Five texts were from Norway, three from Finland, one from Denmark, and none were from Iceland.

**Figure 2.**
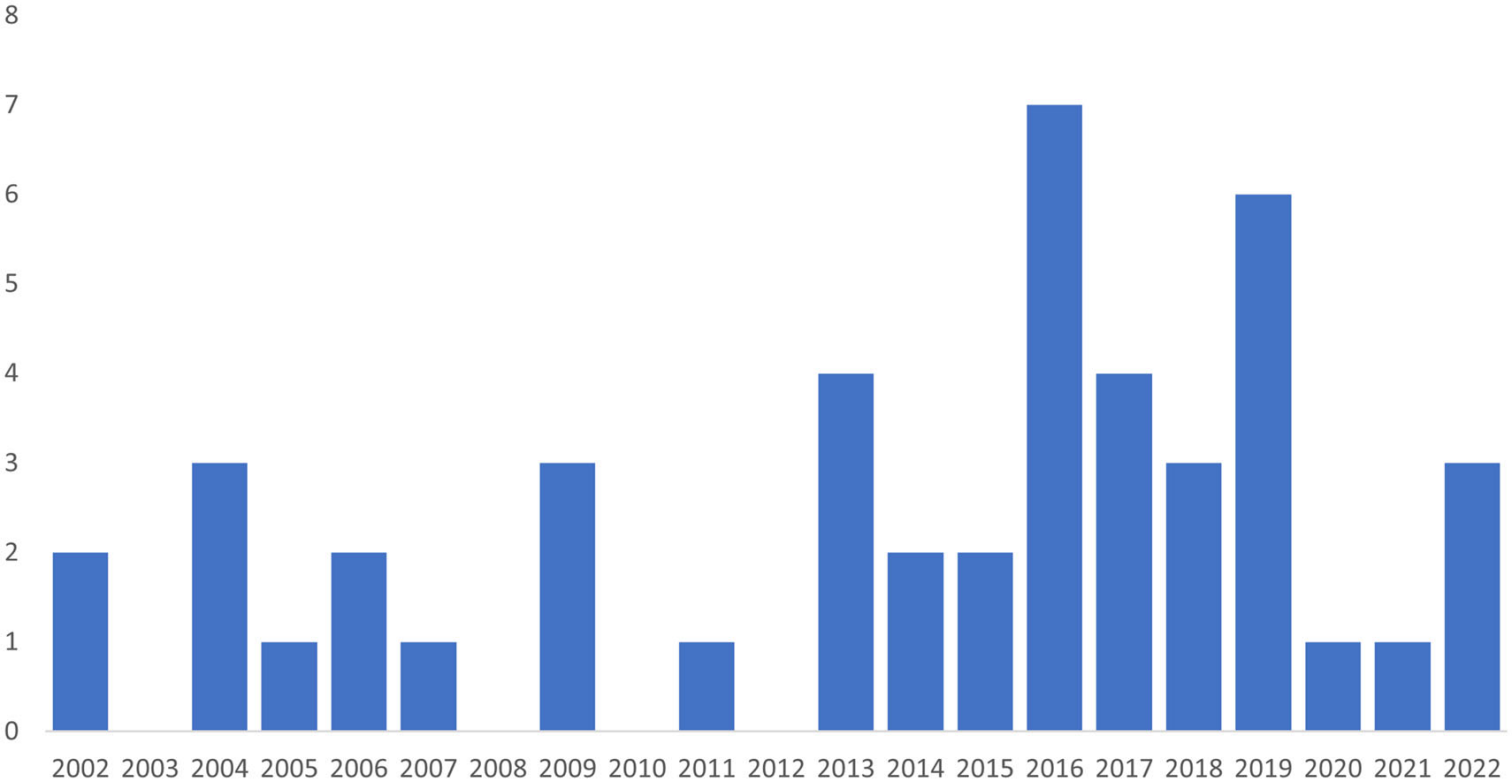
Number of publications by publication year.

While 13 of the texts were published in the first decade of the study period (2002–2011), 33 texts were published during the last decade of the study period (Figure 2). This indicates an increase in published texts concerning LGBT people and services over the 20-year study period.

Two of the journal articles and one of the doctoral theses were studies on documents that direct education, 16 studies looked at healthcare students’/providers’ attitudes to, or knowledge of, LGBT issues, and 23 texts focused on LGBT peoples’ needs, their expectations of and/or experiences with healthcare services^4^ (Table 2). Four texts were policy-oriented: more specifically, these papers discussed the principles and practice of the delivery of healthcare to persons with transgender experiences [13–16]^5^.

**Table 2.** Numeric summary of focus, methodology and country of the included texts.

Thematic focus	Documents that direct education	Students or providers’ attitudes or knowledge	LGBT people and partners’ needs, expectations and/or experiences	Policy	
	*n* = 3 [38,47,48]	*n* = 16 [18–20,22–25,33,39–41,52,55–58]	*n* = 23 [17,21,26–32,34–37,42–46,49–51,53,54]	*n* = 4 [13–16]	
Methodology	Qualitative	Quantitative and qualitative	Quantitative	Quantitative population-based sample	
	*n* = 35 [13–16,18–22,26–30,32–35,37,38,42,43,45–52,54–58]	*n* = 3 [24,31,36]	*n* = 8 [17,23,25,39–41,44,53]	*n* = 2 [17,44]	
Sexual /gender minorities	T	LGBT	LGB		
	*n* = 14 [13–16,18,22,27,29,30,37,51–54]	*n* = 17[19,21,23,25, 26,32,36,38,42,44, 45,47,49,55–58]	*n* = 15 [17,24,28,31,33–35,39–41,43,46,48,50,55]		
Texts per country	Sweden	Norway	Finland	Denmark	Iceland
	*n* = 37 [14,16–20,22–24,26–34,36–38,40,41,43–45,47,49–58]	*n* = 5 [13,35,42,46,48]	*n* = 3 [15,21,39]	*n* = 1 [25]	*n* = 0
Texts about *older* LGBT adults per country	Sweden	Norway	Finland	Denmark	Iceland
	*n* = 10 [22,26,27,32,34,36,45, 49,54,58]	*n* = 2 [42,46]	*n* = 1 [21]		

The majority of the included journal articles and theses were based on qualitative methods. Three articles/theses were based on a combination of quantitative and qualitative methods. Eight papers were based on quantitative methods. Of these, six were based on relatively small or self-selected samples, while two of them were population based.

Fourteen studies focused exclusively on gender minorities, 15 exclusively on sexual minorities, and 17 on both groups. The texts focusing on both groups were centred around sexual minorities. Some texts had LGBT issues as a main topic, while in other texts LGBT issues were only briefly mentioned. Few people belonging to other minorities such as ethnic minorities and few bisexual people participated in the qualitative studies, and the experiences of people belonging to ethnic minorities and bisexual people were almost absent in the included studies. As mentioned earlier, 13 texts (six papers, two PhD theses, and five book chapters) were exclusively about older age groups. Of these, 10 were from Sweden, two from Norway and one from Finland.

## What is known about the provision of healthcare services to older LGBT adults

### Lack of knowledge; cis- and heteronormativity; varying attitudes; and little more than rhetoric in education

In a study based on a large population-based sample, Gustafsson et al. concluded that inequalities in health between sexual minorities and the majority population are explained not only by factors such as harassment and discrimination but also by financial disadvantage, discrimination on the labour market and unmet healthcare needs [17]. Many of the other studies included in our review suggest explanations to why healthcare services are of insufficient quality, resulting in unmet healthcare needs. Some central explanations are lack of knowledge, cis- and heteronormativity, and negative attitudes among healthcare providers.

Studies concerning healthcare students and providers described insufficient knowledge and a wish for more knowledge about LGBT people and their health and care needs [18–25]. Moreover, studies about LGBT service receivers described a lack of knowledge among healthcare providers as a considerable challenge [16,26–30]. Among the topics in which healthcare providers had insufficient knowledge, was the difference between gender identity and sexual orientation [18,27], and the use of terminology, herein the use of preferred pronouns [22]. In addition, knowledge about *older* LGBT adults is lacking, since knowledge on LGBT issues is derived from societal discourses and media, where younger generations are in focus [22]. Moreover, the lack of knowledge is particularly prominent when patients define themselves outside of the gender dichotomy [18]. Studies showed that lack of knowledge among healthcare providers lead to transgender people having to teach their providers [27,29]. This is experienced as demanding and tiresome, contributing to a feeling of estrangement [29], and leads to worries concerning future contact with healthcare providers and whether the providers have sufficient competence to address their medical needs [26].

Several of the included studies described that cis- and/or heteronormative^6^ discourses dominated in healthcare services [13,22,26–29,31–37]. The expectation of heterosexuality was communicated verbally, in waiting rooms and other public areas, through brochures and other information materials [28,31]. However, studies also gave examples of providers which used neutral language and providers who questioned their own gender-binary thinking [24,38].

In some studies, the attitudes of students and/or healthcare providers towards homosexuality were explored. These studies suggested that a majority of students and providers expressed positive attitudes towards homosexuality, while a minority expressed negative attitudes – some of which were very negative [31,39–41]. Møllerop emphasized that the attitudes of providers may influence their service provision and how LGBT people are met [42]. In the included studies, examples of both positive and negative attitudes and encounters were given [26,32,43]. Møllerop added that the attitudes of other patients must also be taken into consideration and that LGBT people risk being isolated in institutions due to the negative attitudes of other patients [42].

Two studies indicated that negative attitudes or perceived discrimination led LGBT people to postpone or refrain from seeking healthcare [37,44]. In addition, LGBT people worried about what attitudes they would meet in future encounters [26,27]. Studies have also described insecurity regarding ‘coming out’ to healthcare providers since LGBT people were unsure of their attitudes and how they would treat them [31,43,45]. Löf and Olaison added that older LGBT adults are careful when choosing which healthcare providers they disclose their LGBT identity to [45], while Malterud and Bjørkman described the process of considering whether to disclose or conceal, and how concealment is a continuous ‘piece of work’, carried out for the sake of security or convenience, implying a broad range of personal costs [35]. Two studies added some insights on reasons why concealment implies personal cost: It is important to be able to tell your life story and have your relationship recognized, and concealment restrains honesty and authenticity [26,45]. Kristiansen adds that concealment previously often was strictly necessary for LGBT people, since LGBT identities have been criminalized and pathologized, and that some people who have always concealed their sexual orientation, continue this practice in older age [46], something which may contribute to making non-heterosexuality among older adults ‘invisible’ to healthcare providers.

Three of the included texts focused on documents that directed the education of healthcare personnel [38,47,48]. Giertsen found that sexuality was addressed to a low degree and heteronormativity seldom problematized in Norwegian social work bachelor’s programs curriculum [48]. Tengelin et al. described documents regulating Swedish nursing education as both using political correct rhetoric and conveying outdated views on identity and normality, drawing a line between ‘us’ and ‘them’, positioning the nursing student as the ‘normal’ person who needed to learn about ‘other’ sexualities [38,47]. Tengelin et al. concluded that the politically correct language in documents that direct nursing education is little more than rhetoric [38,47].

### Neglected partners, the expectation of heterosexual asexual older adults, varying social networks and the importance of structural factors

Some of the included texts dealt with partners and their experiences with healthcare service encounters [26,28,31,43]. Studies described partners who were not treated as ‘true’ partners, experienced negative communication in encounters, were neglected by nursing staff, were not given information, and/or were excluded from decisions [26,28,31,42,43]. Röndahl et al. added that partners of LGB patients felt that they were treated worse as partners than as patients [28].

A small number of studies directed attention towards older LGBT people and sexual practices [32,46,49,50]. Some of these studies described a tendency to see older people as heterosexual and to expect sexual practices such as (hetero)sexual intercourse [32,49,50]. However, this expectation may lead to feelings of estrangement for people who see sexual practice as something more and/or something else [49]. Danemalm et al. added that bodily changes can mean different things in relation to sex for different groups [50]. They interviewed cancer survivors and described how gay men’s narratives about sexual changes circle around the same bodily changes as heterosexual men. However, the bodily changes meant different things to gay men than to heterosexual men. They conclude that one should be more attentive to how bodily changes and side effects of treatment are connected to identity and social relations [50]. In addition, Stålbrand describe that there is a tendency to see older people as not only heterosexual but also asexual [49]. However, although some older people have discontinued their sexual practices, others are sexually active, and people who live in nursing homes may experience that sexual practices are prevented by rigid institutional routines and by personnel who enter the room without waiting for an answer [32,49].

A few qualitative studies discussed older LGBT adults’ social networks [26,27,42]. According to Møllerop and Siverskog some LGBT people have strong and lasting friendships that can be referred to as their ‘chosen family’ [26,42]. However, Siverskog adds that while some of her LGBT interviewees had large social networks, others did not, and the majority of the older transgender adults she interviewed had small social networks, which increased their dependence on public healthcare services [26].

Some studies directed attention towards the fact that institutional and structural factors influence older LGBT adults’ healthcare experiences [22,27,32,34,36,42,45]. Studies emphasized that as long as measures at the organizational level are lacking, older LGBT adults are at the mercy of individual healthcare workers and their widely varying attitudes and competence [27,45]. Siverskog added that constantly meeting different providers who have insufficient time to talk, inhibits communication and hinders openness [32]. One specific institutional factor that was discussed in two studies was LGBT-specific elderly housing [34,36]. LGBT people who discussed specific housing for senior LGBT people, held the opinion that specific housing could represent segregation – in conflict with the ideology of an inclusive and open society that has been central to the LGBT movement [36]. On the other hand, as long as LGBT people experience discrimination and having their way of living questioned, LGBT-specific housing for older people may represent a safe place and have advantages [34].

### Criticism of gender affirming medical treatment

Unlike the other subgroups under the LGBT umbrella, a proportion of people within the group of people with trans experiences seek healthcare in the form of gender-affirming treatment, such as hormone treatment and sex-confirming surgery. Four papers dealt with healthcare policy related to gender-affirming treatment [13–16]. These studies indicate that in Sweden, Norway and Finland, the systems of giving access to sex-confirming surgery have similarities, as the process in these countries is based on care-seekers sending an application, and a centralized approval system. Applications are granted only after a thorough approval process carried out by evaluators, often psychiatric or psychological experts, focusing on the mental health and decision-making ability of the care-seeker [13–16]. Both the roles and discourses held by these evaluators, the guidelines regulating gender-affirming treatment, along with other aspects of systems of approval are criticized in the included studies [13–16,27,30,51–53].

According to studies, evaluators of sex-reassignment surgery applications hold a gatekeeping function: they are the ones who have to be convinced of the legitimate sincerity of the applicant’s wish/need for surgery, as well as their decision-making ability [14,16]. This gatekeeping function is found to undermine the care-seekers ability to attain self-determination [14], and the process of accessing gender-confirming healthcare is found to be contributing to ill health among transgender people going through this process [16]. Rosqvist et al. added that evaluators of applications for gender reassignment surgery are influenced by a developmental-psychological discourse which casts transgenderism as an identity crisis, and imposes certain limitations in available positions for transgender people seeking gender-reaffirming medical treatment, as within this discourse there is a demand for maturity and having gone through all the steps in an expected identity development process [52].

Across studies focusing on policy directed at healthcare for transgender people, there is a common finding that guidelines for trans-specific healthcare in Sweden, Norway and Finland rest on implicit cisgender assumptions and an understanding of gender identity as something binary and stable [13–16]. A study critically analyzing Swedish guidelines for trans-specific care, found that these guidelines (issued in 2015) construct gender dysphoria as psychopathology [14].

People with trans experiences have described both gender binary and heteronormative expectations in transgender care, along with the feelings of having to adhere to these expectations to receive treatment [29]. Consequently, they conceal their sexual orientation, partner, and/or their nonbinary identity if these are not in line with expectations [29]. Sometimes care-seekers seek to bypass the abovementioned gatekeeping function of evaluators by developing strategic narratives and actions [16]. Some feel the need to ‘adapt stories’ (of identity and life) and ‘say the right things’ to fulfil certain diagnostic criteria and fit a certain template in order to receive care [16,29,30]. Linander has added that trans-specific healthcare is experienced as difficult to navigate, due to long waiting times, lack of knowledge among healthcare personnel, lack of support, and relationships of dependency between healthcare providers and users [16].

Four texts focused on *older* transgender people [22,26,27,54]. Among these are three texts by Siverskog [26,27,54]. Here, she points out that older transgender people face the specific challenges of both having an ageing body which limits the ability to undergo gender-reaffirming surgery [26,27], and facing ageist attitudes during the process of transitioning [54]. Siverskog added that older transgender adults report a history of negative experiences from previous encounters with healthcare services, including having to answer very personal questions to be considered for gender-affirming treatment, and this lead to a lack of trust in healthcare services [27].

### Implications for education of care professionals and for delivery of services

Several studies suggest increased attention on LGBT issues in curriculum and education, and a more inclusive healthcare curriculum [23–25,47,48,55]. Both Giertsen and Tengelin et al. suggest that the documents that direct education of future healthcare providers should be revised, and they added that, instead of presenting non-heterosexuality as a deviance, there is a need to interrupt heteronormative thinking and introduce norm-critical perspectives into healthcare education [38,47,48]. Introducing norm-critical approaches both in healthcare education and practice was also recommended in other papers [22,32,47,49,50,56,57], and Lundberg et al. have elaborated that norm-critical approaches interrogate the process of oppression itself and concentrate on groups with privileges and assumed norms [56]. This contrasts with perspectives building on tolerance and the rights of LGBT individuals, which are perspectives that leads to power and privilege for the tolerating groups [56]. In addition, Tholin et al. has argued for the need for the integration of content covering the special circumstances and health needs of transgender patients into the curriculum of nursing and medical schools [37].

Studies also have described a need for training healthcare professionals in LGBT issues [16,21,27,30,37,40]. It is emphasized that healthcare professionals should avoid cis- and heteronormativity and a dichotomous understanding of gender [27,29,42,45]. Furthermore, they should reflect on their own behavior and be aware of their own attitudes and norms [18,43], and they should strive to use non-heteronormative, gender-neutral and open language, including preferred pronouns and names [24,28,31,32,42,58]. The use of affirming visual signs in healthcare settings is also recommended in studies [27,28,30,45]. In some studies, the marginalization history embodied by older LGBT adults has been thematized, with emphasis on the importance of awareness and knowledge of this history, among others since it influences peoples’ degree of openness [27,42,45]. Siverskog has added the importance of being aware that LGBT people have very various experiences [27].

In some studies, the meaning of equal treatment is given attention [22,45]. It is argued that the vision of treating everyone equally, in reality, could mean treating everyone in the same way. However, providers are part of the general society. Therefore, it is likely that providers bring normative ideas, including heteronormativity, into practice. Consequently, treating everyone in the same way, while relying on taken-for granted norms, can easily lead to discrimination and should be avoided [22,45].

Gustafsson et al. have concluded that since inequalities in health between LGBT people and the majority population is not only a result of discrimination, but also economic disadvantage, discrimination on the labour market and poorer access to healthcare, greater consideration should be given to these factors [17]. Moreover, authors writing about LGBT issues and healthcare services have emphasised the importance of implementing measures on the meso- and macro level [22,32,36,42]. Smolle et al. have elaborated that, to be able to develop good reflective practices and norm-critical approaches, certain institutional and organizational prerequisites are required [22]. Møllerop has added that institutions should have structural and permanent systems for handling situations where individuals have been or are at risk of becoming isolated as a result of other residents’ negative attitudes [42], while Kottorp et al. have suggested that LGBT-profiled senior housing can be a good alternative for some LGBT people, as it provides safe places [36].

Regarding implications for gender-affirming treatment, Linander et al. suggest trans-specific care practices are ripe for changes. Their main point is that trans-specific healthcare should change in a direction that assures individuals identified in a non-binary way receive the same quality of care as those identifying in binary ways, regardless of geographical location [51]. At the policy level (in a Swedish context) studies have indicated the need for new guidelines, as well as a need to consider alternative models to the currently existing gatekeeping model of evaluators. More specifically, they have suggested that one such alternative model could be informed consent models, in which care-seekers are given the opportunity to begin on hormones without prior mental health evaluation [14,38]. Furthermore, Linander et al. has highlighted that more informed healthcare providers, coupled with increased awareness and critical thinking concerning gender and cisnormativity, could minimize the reconstruction of cisnormative norms within healthcare, thereby improving the quality of trans-specific healthcare services [51]. Linander et al. have also flagged the need for trans-specific healthcare to undergo enhancements, in such a way that gender-confirming procedures become more accessible and practices more affirming, while leaving increased space for self-determination, more patient- and individual-focused care, and, not least, giving trans-specific healthcare more prioritization, increasing efficiency and reducing waiting times [16,51].

## Discussion

In this study we have aimed to examine what is known about the provision of healthcare services to older LGBT adults in the Nordic countries; identify knowledge gaps; map implications of this research for the education of healthcare professionals and for delivery of services to older LGBT adults; and to identify key future research priorities.

Several of the included studies showed that healthcare personnel lack sufficient and adequate knowledge on LGBT issues. In particular, knowledge about *older* LGBT adults, and individuals who identify as non-binary, is lacking. In addition, LGBT people describe being met with cis- and heteronormativity in healthcare encounters, and studies analyzing documents that direct healthcare educations, conclude that LGBT issues are scarcely mentioned. Studies on attitudes towards homosexuality conclude that some providers hold negative attitudes, and older LGBT people worry about how they will be met when contacting healthcare services, and some postpone or refrain from seeking healthcare. Access to gender-affirming medical treatment for transgender individuals is managed by gatekeepers who may be influenced by pathologizing discourses and a binary way of understanding gender. Thus, transgender people often adhere to the gatekeeper’s expectations and strategically ‘say the right thing’ to receive treatment. Consequently, studies suggest increased attention on LGBT issues in the education of healthcare students, training of healthcare professionals, as well as implementing measures on institutional levels to ensure inclusive services. Furthermore, studies suggest that trans-specific healthcare practices should be changed in a way that mean individuals who identify as non-binary would receive the same quality of care as individuals who identify in a binary way.

The findings that healthcare providers lack knowledge, heteronormativity is communicated in healthcare encounters, and that older LGBT adults worry about how they will be met and refrain from seeking healthcare, are in line with findings in four review articles which included papers mainly from the USA, Canada and the United Kingdom [2–5]. In a study from Canada, where the lack of knowledge among healthcare personnel is discussed, the authors concluded that insufficient focus on LGBT topics in healthcare education is the most important reason for this lack of knowledge [59]. Thus, the authors argued that LGBT topics to a larger degree should be included in healthcare education [59]. In a study from the USA, the topic of refraining to seek health care because of anticipated discrimination is discussed, and findings showed that 78.6% of older LGBT adults anticipated discrimination in contact with healthcare services [60]. As for older LGBT adults in other countries, the negative attitudes and discrimination that older LGBT people in the Nordic countries experience, can be related to homophobia and transphobia, intersected with ageism [61]. Further, based on intersectionality reasoning, it is reason to believe that factors such as gender, socioeconomic position, race and ethnicity also influence how older LGBT adults are met in healthcare encounters [61]. On the other hand, literature from the Nordic countries about adolescents and younger adults, suggest that lack knowledge about trans issues among healthcare personnel is a general challenge regardless of the patient’s age and other demographic characteristics [62,63].

The concept of ‘epistemic injustice’ may help illuminate the reasons why knowledge about LGBT issues is lacking [64,65]. Fricker has described the term epistemic injustice and has used it to show how the privileged have unfair advantages with regards to forming our collective understandings, while the disadvantaged groups are hermeneutically marginalized [64]. She has exemplified this by describing how powerful groups historically have defined how homosexuality should be understood and interpreted, while homosexual individuals themselves were excluded from presenting their versions. The result was a de facto exclusion of homosexuals participating in the forming of our common understanding of homosexuality [64]. Andersson has added that people tend to communicate with people who belong to the same group as themselves, a factor restricting the development of interpretive resources that enable them to understand other groups. She elaborates further that ‘from the perspective of the advantaged, what the disadvantaged are saying makes no sense because the interpretive resources they have developed (…) are inadequate for comprehending experiences of those from whom they are isolated’ [65, p. 170]. Thus, the lack of understanding of other groups are not necessarily a result of prejudice but could rather be sheer incomprehension [65]. In summary, the lack of understanding and knowledge about LGBT issues can be explained as a result of the fact that certain LGBT groups’ stories have been marginalized in the process of forming our common understandings coupled with a lack of interpretive resources in the mainstream to actually comprehend the stories that, despite marginalization, are being told. The findings in this review suggest that the stories of in particular bisexual individuals and individuals who identify as non-binary, to a low degree, are told and understood, leading to a considerable lack of knowledge about these groups. Fricker’s and Andersson’s advices for reducing epistemic injustice are to strive for proactive and socially aware listening in dialogs with underprivileged groups, universal participation and measures on a structural level to hinder injustice towards underprivileged groups [64,65]. The latter are in line with van der Ros – who suggested that there should be representation from various trans organizations in cases concerning transgender people [13] – and studies that have emphasized the importance of structural factors to enhance the quality of healthcare services for LGBT people [22,32,36,42].

Our systematic literature search yielded few results from Nordic countries besides Sweden. Furthermore, we found few population-based quantitative studies and few studies about older age groups. In addition, bisexual individuals turned out to be a neglected group. Consequently, more research on the abovementioned groups, more quantitative research based on population data, and more research from Nordic countries besides Sweden is needed to fill knowledge gaps. Furthermore, according to Gustafsson et al. LGBT people experience economic disadvantage, and discrimination on the labour market, and greater attention should be given to these factors [17]. In addition, little focus on LGBT topics in healthcare education seem to be a vital cause for lack of knowledge among healthcare personnel [59]. Our search revealed few studies focusing on healthcare education and the regulation of such education. Thus, a key future research priority should be on healthcare education, focusing on how and to what extent LGBT-related topics are presently presented and how they could be included in the future.

Our search resulted in 46 included texts. However, only 13 of these were specifically about older age groups. Therefore, many of the findings presented here are, to a large degree, based on data from younger adults. We chose to include studies published in and after 2002, and the attitudes towards LGBT people, the quality of healthcare services and documents that direct education may to some extents have changed since the studies were published. In addition, we did not include studies in Finnish and Icelandic languages, or grey literature. While, the inclusion of grey literature in Finnish and Icelandic languages would have added more information about older LGBT adults and healthcare services, a literature review on LGBT ageing on behalf of the Nordic Council of Ministers showed that there were no scientific papers published in peer-reviewed journals or approved PhD theses in Finnish and Icelandic languages [66].

## Conclusion

Our systematic literature search revealed a scarcity of research on older LGBT adults in conjunction with healthcare services. Furthermore, we found few population-based quantitative studies, few studies from Nordic countries besides Sweden, few studies on healthcare education, and bisexual people represented a neglected group. Thus, more research on the abovementioned topics, more quantitative research based on population data, and more research from Nordic countries besides Sweden is needed.

The included studies showed a lack of knowledge on LGBT issues among healthcare personnel. In particular, knowledge about older LGBT adults, and individuals who identify as non-binary, was lacking. Older LGBT adults who access health and care services are met with cis- and heteronormative expectations, and individuals who seek trans-specific treatment are met by gatekeepers in a centralized approval system, who may be subscribing to pathologizing, linear, and binary discourses on gender. Accordingly, studies suggest increasing attention on LGBT issues in education; training for healthcare professionals; institutional level measures; proactive and socially aware dialogues; and ensuring that transgender individuals who identify as non-binary receive the same quality of care as others.

## Notes

1 Old(er) and young(er) are relative notions. In line with United Nation’s definition, for the purposes of this study, we chose to define an ’older person’ as an individual 60 years of age or more [1].

2 LGBT is an abbreviation used for sexual and gender minorities. Other abbreviations, such as LGBTQ2+, are also used, where LGBTQ2+ stands for lesbian, gay, bisexual, transgender, queer, and two-spirited. The plus sign signifies several other sexual and gender minority identities.

3 The searches were carried out the following dates in 2022: Svemed February 11; Sociological Abstracts March 22; Nora Open March 23; MEDLINE March 24; Web of Science April 1; Cinahl April 1; Diva-portalen April 4; Proquest Dissertation April 4; Google Scholar May 6.

4 Some of the texts that focused on LGBT people were also about providers.

5 One of the texts that focused on policy, also focused on receivers.

6 Cisnormativity is the assumption that all people identify with the legal gender assigned to them at birth, while heteronormativity is the notion that being heterosexual is the default sexual orientation, and heteronormativity also include a binary gender model.
